# Clozapine-Associated Neutropenia among People on Clozapine in Japan: A Nationally Representative Retrospective Cohort Study

**DOI:** 10.1093/schbul/sbag087

**Published:** 2026-05-29

**Authors:** Mike Trott, Korinne Northwood, Takeo Hata, Keiichiro Nishida, Dan Siskind, Tetsufumi Kanazawa

**Affiliations:** Addiction and Mental Health Service, Metro South Health, Woolloongabba, QLD 4102, Australia; Faculty of Health, Medicine and Behavioural Science, University of Queensland, Brisbane, QLD 4067, Australia; Physical and Mental Health Stream, Queensland Centre for Mental Health Research, Wacol, QLD 4076, Australia; Addiction and Mental Health Service, Metro South Health, Woolloongabba, QLD 4102, Australia; Faculty of Health, Medicine and Behavioural Science, University of Queensland, Brisbane, QLD 4067, Australia; Department of Pharmacy, Osaka Medical and Pharmaceutical University Hospital, Takatsuki, Osaka 569-8686, Japan; Department of Medical Informatics, Osaka Medical and Pharmaceutical University, Takatsuki, Osaka 569-8686, Japan; Department of Neuropsychiatry, Osaka Medical and Pharmaceutical University, Takatsuki, Osaka 569-8686, Japan; Addiction and Mental Health Service, Metro South Health, Woolloongabba, QLD 4102, Australia; Faculty of Health, Medicine and Behavioural Science, University of Queensland, Brisbane, QLD 4067, Australia; Physical and Mental Health Stream, Queensland Centre for Mental Health Research, Wacol, QLD 4076, Australia; Department of Neuropsychiatry, Osaka Medical and Pharmaceutical University, Takatsuki, Osaka 569-8686, Japan

**Keywords:** schizophrenia, clozapine, neutropenia, Japan

## Abstract

**Background and Hypothesis:**

Clozapine is the most effective antipsychotic for treatment-resistant schizophrenia, but use is constrained by potentially life-threatening neutropenia and mandatory hematological monitoring. Evidence from Western cohorts suggests risk is concentrated early in treatment, yet data from Japan where monitoring is stringent, remain limited.

**Study Design:**

Retrospective study including all patients prescribed clozapine from July 2009-January 2020 in Japan. Mild neutropenia was defined as absolute neutrophil count (ANC) 1.0-1.5 × 10^9^/L, and serious neutropenia as ANC <1.0 × 10^9^/L. Cumulative incidence of first mild and serious neutropenia was estimated using competing-risks methods. Associations with serious neutropenia were examined using Fine–Gray regression, including 2-week titration rate.

**Study Results:**

The cohort comprised 8263 individuals contributing 764 180 blood tests, with a median follow-up of 102 weeks. Overall, 3.0% patients experienced mild neutropenia and 2.2% had serious neutropenia. Among clozapine-naïve patients, cumulative incidence was 1.8% for mild and 1.6% for serious neutropenia at 18 weeks, rising to 3.1% and 2.5% at 104 weeks, respectively. Faster clozapine titration rate was associated with higher incidence of serious neutropenia (sub-distribution hazard ratio [sHR] 1.22, 95% CI, 1.02-1.45 per 50 mg dosage increase at 2-weeks), as was older age (sHR 1.05, 95% CI, 1.04-1.06), while prior clozapine exposure was associated with lower incidence (sHR 0.27, 95% CI, 0.09-0.86).

**Conclusions:**

In Japan, neutropenia among clozapine-treated patients accrued predominantly within the first 1-2 years, with faster titration in the first 2-weeks being associated with a 22% increase in serious neutropenia risk per 50 mg. These findings support risk-stratified, less intensive monitoring approaches beyond the first 2 years.

## Introduction

Among people with schizophrenia, one-third will not respond to 2 adequate trials of first-line antipsychotic medications.^1^ Among this group of people with treatment resistant schizophrenia,^2^ clozapine is the most effective antipsychotic for reducing positive psychosis symptoms,^3^ psychiatric hospitalizations,^4^ and all-cause mortality.^5^ Its use, however, is constrained globally by the risk of potentially life-threatening neutropenia, which underpins mandatory hematological monitoring schemes in many jurisdictions.^6^ In Japan, for example, monitoring intervals have historically been among the strictest in the world. At the time of clozapine’s introduction in Japan, blood tests were required with a maximum interval of 7 days at weeks 1-2 post initiation, extending to 14 days after 26 weeks.^7^ Although such monitoring is designed to enhance patient safety, it also imposed substantial burden and cost for patients and health systems. As of 2021, regulations were revised with weekly monitoring for the first 6 months, fortnightly in months 7-12, and every 4 weeks after 1 year of stable treatment.^8^ There is a growing body of evidence, however, suggesting that the risk of clozapine-associated neutropenia is highest during early treatment, and decreases substantially over time. For example, a large binational analysis of the Australian and New Zealand Viatris Pharmacovigilance System^9^ showed that 99% of serious neutropenic events occurred within 18 weeks, with negligible risk thereafter, suggesting that current lifelong monitoring requirements may be unnecessarily stringent. Furthermore, several registry based studies have shown that rates of neutropenia are the highest in the first 18 weeks, with decreasing risks across time.^10^^,^^11^

Although ethnic variation in susceptibility to drug-induced neutropenia is well recognized, with benign ethnic neutropenia (BEN) common among individuals of African, Middle Eastern, or certain Asian ancestries,^12^ few studies have investigated how these patterns manifest in East Asian populations, where genetic, dietary, and environmental factors may also influence neutrophil physiology and risk of clozapine-related neutropenia. To address this gap, we analyzed post-marketing pharmacovigilance data from the Japanese national clozapine monitoring system, aiming to (1) determine the epidemiology and timing of neutropenic events among people treated with clozapine in Japan, (2) examine whether risk patterns mirror those observed in Western cohorts, and (3) explore whether ethnicity-linked factors—such as lower baseline neutrophil counts—might partially account for international differences in monitoring thresholds and safety outcomes.

## Methods

### Data Source

We conducted a retrospective cohort analysis using the Clozaril Patient Monitoring Service (CPMS) Centre in Novartis Pharma, Tokyo, Japan. This dataset included anonymized data from everyone who was administered clozapine in Japan from July 31, 2009, through January 26, 2020. The dataset included the date of patient registration, registered medical institution, sex, date of birth, date of examination, white blood cell count, absolute neutrophil count (ANC), blood glucose, hemoglobin A1c (HbA1c), date of prescription, and clozapine dosage. Blood levels of clozapine were not included in the dataset. Consistent with prior publications using this database,^8^^,^^13^ duplicate registrations due to institutional transfer were reconciled to yield only unique individuals for analysis. Full demographic information on the cohort has been reported elsewhere.^13^

### Definitions and Measures

Baseline ANC was defined as the first recorded value per participant in the CPMS dataset, and neutropenic events were classified using absolute neutrophil count thresholds consistent with the US common terminology criteria for adverse events and previous clozapine neutropenia studies.^9^^,^^14^ Specifically, mild neutropenia was defined as 1.0-1.5 × 10^9^/L, serious neutropenia <1.0 × 10^9^/L, and agranulocytosis <0.5 × 10^9^/L.

### Statistical Analysis

Descriptive statistics were used to summarize baseline characteristics. Group differences between patients with and without prior clozapine exposure were tested using χ^2^ tests for categorical variables and Wilcoxon rank-sum tests for continuous variables. Incidence rates of mild and serious neutropenia were expressed as events per 100 person-years. Furthermore, annualized incidence rates per 100 person-years were also calculated for agranulocytosis, mild, and serious neutropenia for year 1, 2 and year 3+, respectively.

All analyses were conducted across 2 groups: patients who were clozapine naïve and patients with previous clozapine exposure, defined using CPMS registration data that flagged previous clozapine exposure. Absolute risk of mild and serious neutropenia was estimated using competing-risks cumulative incidence analysis, with participants followed from clozapine initiation until their first qualifying neutropenic event. Mild neutropenia and serious neutropenia were treated as mutually competing events. Time to event was defined as the interval between clozapine initiation and the first ANC result meeting criteria for either mild or serious neutropenia (due to anticipated small cell counts, agranulocytosis was not considered in this hierarchy and only used in exploratory descriptive analyses). In the competing-risks framework, mild neutropenia was treated as a competing event for serious neutropenia, while participants without an event were censored at their final blood test.

Associations with the cumulative incidence of serious neutropenia were examined using a competing-risks regression analysis and reported as sub-distribution hazard ratios (sHRs). Within the competing-risks framework, mild neutropenia was treated as a competing event for the outcome of serious neutropenia, while participants without neutropenia were censored at their final blood test. Covariates in the model included age, gender, baseline neutrophil count, and prior clozapine exposure (no other variables were available). To examine the potential effects of the rate of clozapine titration, a competing-risks regression analysis was conducted, with clozapine dose at 2 weeks (per 50 mg increase) as an additional co-variate to the primary model (age, gender, baseline neutrophil count, and prior clozapine exposure). To avoid immortal time bias, only people who were event free at 2 weeks were included in the clozapine titration analysis. An exploratory competing-risks regression was also conducted to determine if prior antipsychotic use was a significant co-variate for serious neutropenia.

Furthermore, exploratory cumulative incidence analyses were conducted using agranulocytosis as an outcome.

All analyses were performed in R (version 4.4.0).

### Ethics

This study received an exemption from ethics review, granted by the University of Queensland Human Research Ethics Committee (2024/HE000975).

## Results

A total of 8263 participants were present in the dataset, encompassing 764 180 unique blood tests. The total sample was 54% male, with a mean age at clozapine commencement of 41.6 years (SD = 12.1). The median follow up time was 102 weeks (IQR 30-235), and 94.7% of the sample did not have previous clozapine exposure. Of the total sample, 250 (3.0%) of patients had a mild neutropenic event, 185 (2.2%) had a serious neutropenic event. Furthermore, 84 (1.0%) patients had agranulocytosis. When adjusted for person-years of exposure, the overall neutropenia event rate was 1.9 per 100 person years, 1.1 per 100 person years for a mild neutropenic event, and 0.8 per 100 person years for serious neutropenic events, see Table 1. Among clozapine-naive patients, the rate of any neutropenic event per 100 person years fell from 5.31 (95% CI, 4.75-5.92) in year 1 to 0.85 (95% CI, 0.60-1.16) in year 2, and 0.46 (95% CI, 0.34-0.60) in year 3 and beyond. The corresponding rates for mild neutropenia were 4.44 (95% CI, 3.93-4.99), 0.80 (95% CI, 0.56-1.11), and 0.41 (95% CI, 0.30-0.54) per 100 person-years, and for serious neutropenia were 2.45 (2.07-2.87), 0.27 (0.14-0.47), and 0.16 (0.09-0.25) per 100 person-years. More information can be found in Table S1.

**Table 1:** Demographic Characteristics

		All patients (*n* = 8263)	Patients without previous clozapine exposure (*n* = 7828)	Patients with previous clozapine exposure (*n* = 435)
Unique pathology tests		764 180	732 927	31 253
Gender	Male	4470 (54%)	4227 (54%)	243 (56%)
	Female	3793 (46%)	3601 (46%)	192 (44%)
Prior antipsychotic use^a^	Olanzapine	4650 (60.7%)	4530 (60.7%)	120 (62.8%)
	Risperidone	3801 (49.6%)	3704 (49.6%)	97 (50.8%)
	Aripiprazole	2059 (26.9%)	2012 (26.9%)	47 (24.6%)
	Quetiapine	1525 (19.9%)	1496 (20.0%)	29 (15.2%)
	Blonanserin	1210 (15.8%)	1181 (15.8%)	29 (15.2%)
	Paliperidone	1145 (14.9%)	1116 (14.9%)	29 (15.2%)
	Perospirone	250 (3.3%)	243 (3.3%)	7 (3.7%)
	Typical (first-generation) antipsychotic	766 (10.0%)	744 (10.0%)	22 (11.5%)
	Other atypical antipsychotic	527 (6.9%)	521 (7.0%)	6 (3.1%)
Age at clozapine commencement (years)		41.6 (12.1)	41.4 (12.1)	44.3 (12.7)
Baseline neutrophil counts (×10^9^/L)		3.8 (1.5)	3.8 (1.5)	3.9 (1.6)
Neutropenic events, per 100 person-years	Any event	1.89	1.90	1.80
	Mild	1.09	1.07	1.46
	Serious	0.81	0.82	0.34
Neutropenic events	None	7828 (94.7%)	7409 (94.6%)	419 (96.3%)
	Mild	250 (3.0%)	237 (3.0%)	13 (3.0%)
	Serious (total)	185 (2.2%)	182 (2.3%)	3 (0.7%)
	Agranulocytosis	84 (1.0%)	84 (1.1%)	0 (0.0%)
Time to neutropenic event, week^b^	Mild	14.4 (6, 51.8)	15.1 (6, 52.3)	10.3 (6.7, 22)
	Serious	13 (8.9, 31.6)	13 (8.9, 31)	18.6 (13.6, 43.4)
Total cohort follow-up time, weeks^b^		102 (30, 235)	104 (30, 239)	69 (25, 156)

^a^Percentages derived from patients with non-missing prior antipsychotic use (n = 7,659).

^b^Data presented as median (IQR).

**Figure 1:**
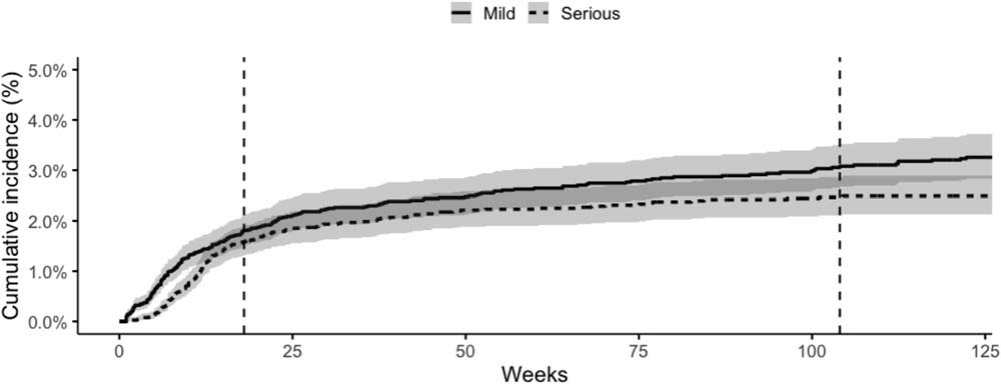
Cumulative Incidence of Neutropenia in Clozapine naïve Participants. The first dotted line represents 18 weeks. The second dotted line represents 104 weeks.

**Figure 2:**
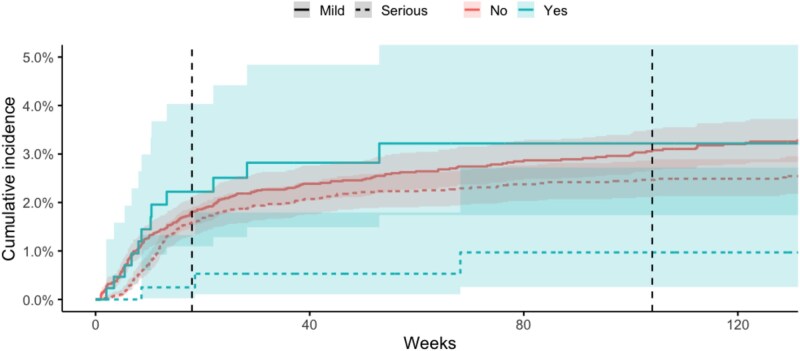
Cumulative Incidence of Neutropenia in Clozapine naïve Versus Non-clozapine naïve Participants. The first dotted line represents 18 weeks. The second dotted line represents 104 weeks.

In people who were clozapine naïve, the cumulative incidence of mild neutropenia was 1.8% (95% CI, 1.5-2.1) at 18 weeks and 3.1% at 104 weeks (95% CI, 2.7-3.5), with incidence plateauing after 104 weeks. The cumulative incidence of serious neutropenia was 1.6% (95% CI, 1.3-1.9) at 18 weeks and 2.5% (95% CI, 2.1-2.9) after 104 weeks, see Figure 1 and Tables S2 and S3. In people who had previously been exposed to clozapine, the cumulative incidence of mild neutropenia was 2.2% (95% CI, 1.1-4.0) at 18 weeks and 3.2% (95% CI, 1.7-5.4) by 104 weeks. The cumulative incidence of serious neutropenia in people previously exposed to clozapine was 0.3% (95% CI, 0.0-1.3) at 18 weeks and 1.0% (95% CI, 0.3-2.7) at 104 weeks, see Tables S4 and S5. When comparing clozapine naïve versus previous clozapine exposure, there were no significant differences in the cumulative incidence of mild neutropenia (*P* = .74), however serious neutropenia was significantly higher among people who were clozapine naïve (*P* = .036), see Figure 2. Exploratory analysis of the cumulative incidence of agranulocytosis yielded an incidence of 0.8% (0.6%-1.1%) at 18 weeks and 1.2% (95% CI, 0.9-1.4) at 104 weeks, see Figure S2.

The competing-risks regression model showed that increasing age was associated with a higher cumulative incidence of serious neutropenia (sHR per year 1.05, 95% CI, 1.04-1.06). Prior clozapine exposure was also associated with a lower cumulative incidence of serious neutropenia (sHR 0.27, 95% CI, 0.09-0.86). Gender and baseline neutrophil count were not significantly associated with risk, see Table 2. For the clozapine titration rate analysis (*n* = 8070), higher clozapine dose at 2 weeks was associated with higher incidence of serious neutropenia in adjusted models (sHR 1.22 per 50 mg increase, 95% CI, 1.02-1.45), with no association found in mild neutropenia, see Table 3 and Table S6. Exploratory analyses showed that the number of prior antipsychotic medications (n = 7460) was not significantly associated with serious neutropenia, see Table S7.

**Table 2:** Competing Risks Regression Analysis for Serious Neutropenia

Covariate	sHR (95% CI)	*P*-value
Age (years)	1.05 (1.04-1.06)	<.001
Gender (male as the reference)	0.87 (0.65-1.16)	.320
Baseline neutrophil count	1.00 (1.00-1.00)	.110
Previous clozapine exposure (reference group: naïve)	0.27 (0.09-0.86)	.026

**Table 3:** Competing Risks Regression Analysis for Serious Neutropenia with Clozapine Titration Rate as a Covariate

Covariate	sHR (95% CI)	*P*-value
Age (years)	1.05 (1.04-1.07)	<.001
Gender (male as the reference)	1.14 (0.85-1.53)	.380
Baseline neutrophil count	1.00 (1.00-1.00)	.230
Previous clozapine exposure (reference group: naïve)	0.29 (0.09-0.91)	.035
Clozapine titration rate (per 50 mg increase at 2 weeks)	1.22 (1.02-1.45)	.027

## Discussion

This nationally representative cohort study from Japan aimed to determine the rates of mild and serious neutropenic events in clozapine users. Results showed that the risk of both mild and serious neutropenia, while clinically important, was concentrated in the early weeks post-clozapine initiation, with lower rates when accounting for only clozapine related serious neutropenia, with plateauing after 1-2 years. These findings align with growing international evidence suggesting that the temporal risk profile of clozapine-associated neutropenia is front-loaded, raising questions about the necessity of lifelong hematological monitoring.

Our results closely mirror those reported in Western cohorts, despite Japan operating one of the most conservative monitoring frameworks globally. Registry-based studies from Australia, New Zealand,^9^ the United Kingdom,^15^ the United States,^10^ Finland,^16^ and Chile^17^ consistently demonstrate that the majority of serious neutropenic events occur within the first 18-26 weeks of treatment, with reducing incidence thereafter. For example, Northwood et al.^9^ reported an absolute rate of mild neutropenia of 0.9 per 100 person-years, similar to our finding of 1.1 per 100 person-years. Our rate of serious neutropenia (0.8 per 100 person-years) was higher than Northwood et al.’s (0.1–0.2), although their estimates were stratified by clozapine cessation, whereas our dataset did not permit this. Furthermore, Alvir et al.^10^ reported cumulative incidence rates of 0.8% and 0.9% after 12 and 18 months, respectively, with our findings being broadly comparable. The present findings extend this international evidence to an East Asian population, suggesting that the early-risk pattern of clozapine-associated neutropenia is generally robust across different regulatory environments, prescribing practices, and population characteristics. Although the absolute event rates of mild neutropenia, serious neutropenia, and agranulocytosis are uncommon at the individual patient level, the non-zero rates indicate clinical significance that continue to justify hematological surveillance in some form. Lifelong intensive hematological surveillance, however, may yield diminishing returns beyond earlier treatment periods. While Japan’s conservative thresholds and 2-weekly long-term monitoring were designed to maximize safety following clozapine’s market introduction, our data suggest that such intensity may not translate into proportionately greater protection against later-onset serious neutropenia.

Results also found that increasing age was associated with serious neutropenia, with risk increasing incrementally with each additional year at clozapine initiation. This finding is in keeping with prior observational studies and may reflect age-related changes in bone marrow reserve, comorbidity burden, or polypharmacy.^10^ In contrast, prior clozapine exposure was associated with a significantly lower cumulative incidence of serious neutropenia, suggesting that individuals who tolerate an initial course of clozapine represent a substantially lower-risk group, supporting potential differentiated monitoring strategies based on treatment history. This finding was different from prior analyses of the same database examining predictors of treatment discontinuation, that reported that previous clozapine exposure was associated with increased likelihood of discontinuation.^18^ Clozapine discontinuation, however, is a composite clinical outcome influenced by clinician and patient factors, whereas the present analysis examined the cumulative incidence of objectively assessed defined serious neutropenia. Furthermore, the analyses methods differed, which may also explain apparent directional differences. Results also showed that baseline ANC was not independently associated with serious neutropenia risk in adjusted analyses. Although ethnic variation in neutrophil physiology is well recognized, including the presence of BEN in some populations, our findings suggest that lower baseline neutrophil counts alone may not be a reliable indicator of clinically significant neutropenic events within the Japanese context. Conversely, previous analyses of the same cohort found that lower baseline white cell counts (<6000/mm^3^) were associated with treatment discontinuation.^18^ Considering the differences found between previous analyses and the present study, it appears that baseline hematological factors may influence clinical management decisions more strongly than they predict subsequent neutropenic events. Furthermore, in the clozapine titration analysis, higher clozapine dose at 2 weeks was independently associated with an increased hazard of serious neutropenia, which is consistent with current Japanese prescribing guidance recommending gradual titration during the initial weeks of treatment, and suggests that even where monitoring durations may be shortened for stable patients, early titration practice remains a potentially modifiable determinant of risk. Previous studies have shown that rapid clozapine titration is associated with clozapine-related fever^19^ and myocarditis,^20^ however, to our knowledge this is the first large cohort study to report these findings. Further research is therefore warranted.

Taken together, these findings have important implications for the future evolution of clozapine monitoring policy. Internationally, regulatory agencies and expert consensus groups have begun to reconsider the necessity of lifelong frequent blood monitoring, particularly in patients who remain stable beyond one to 2 years of treatment.^21^^,^^22^ Of note, the U.S. Food and Drug Administration has eliminated the Clozapine Risk Evaluation and Mitigation Strategy program, reducing the reporting burden for clozapine prescribers and patients,^23^ and the European Medicines Agency has also reduced the monitoring for ANC for people on clozapine after one year,^24^ as well as the 2021 reduction in burden in Japan.^8^ By the third year of clozapine treatment, clozapine-naive patients had an approximately 15× lower incidence for serious neutropenia, and 70× lower incidence for agranulocytosis than Year 1 rates, with only 2 agranulocytosis events observed across 11 361 person-years of follow-up beyond Year 2. Our results provide the first population-level evidence from Japan that late-onset serious neutropenia occurs at substantially lower rates than during early treatment, reinforcing the argument for risk-stratified, less-intensive monitoring across time, while recognizing that low-level risk persists and that continued surveillance remains warranted.

Although this study has several strengths, including comprehensive national coverage, large sample size, and longitudinal blood testing data, several limitations warrant consideration. First, we did not have access to detailed clinical information, including ethnicity, smoking status, comorbidities, and clozapine plasma concentrations, all of which may influence neutropenia risk. Second, due to small cell counts, we were unable to conduct regression analyses distinguishing clozapine-related from unrelated serious neutropenia or agranulocytosis. Third, causality cannot be inferred from observational pharmacovigilance data. Fourth, the conservative monitoring thresholds in Japan may limit direct comparability with jurisdictions using different ANC cut-offs. Finally, the CPMS dataset only captures patients who have been initiated on clozapine and does not include comparator antipsychotic cohorts. We are therefore unable to estimate clozapine-attributable risk relative to other antipsychotic exposures within this dataset.

In conclusion, this national study demonstrates that neutropenia in clozapine users in Japan, while clinically non-trivial, occurs predominantly early in treatment, and mirrors temporal risk patterns observed in other representative cohorts internationally. These findings support consideration of risk-stratified, less-intensive monitoring approaches beyond the first 1-2 years, while recognizing that low-level risk persists and that continued hematological surveillance remains warranted. Considered policy reforms informed by real-world data such as these, may help reduce barriers to clozapine access while maintaining high standards of patient safety.

## Supplementary Material

Supplementary_sbag087
