# Supplementary material for: Clozapine-Associated Neutropenia among People on Clozapine in Japan: A Nationally Representative Retrospective Cohort Study
Source: Schizophr Bull. 2026 May 29;52(3):sbag087. doi: 10.1093/schbul/sbag087 (PMC13220260; doi:10.1093/schbul/sbag087)
Supplement: Supplementary_sbag087 [file supplementary_sbag087.docx]

**Supplementary Figure 1: Cumulative incidence analysis for serious neutropenia comparing serious neutropenia related vs unrelated to clozapine use in people without prior clozapine exposure.**

**
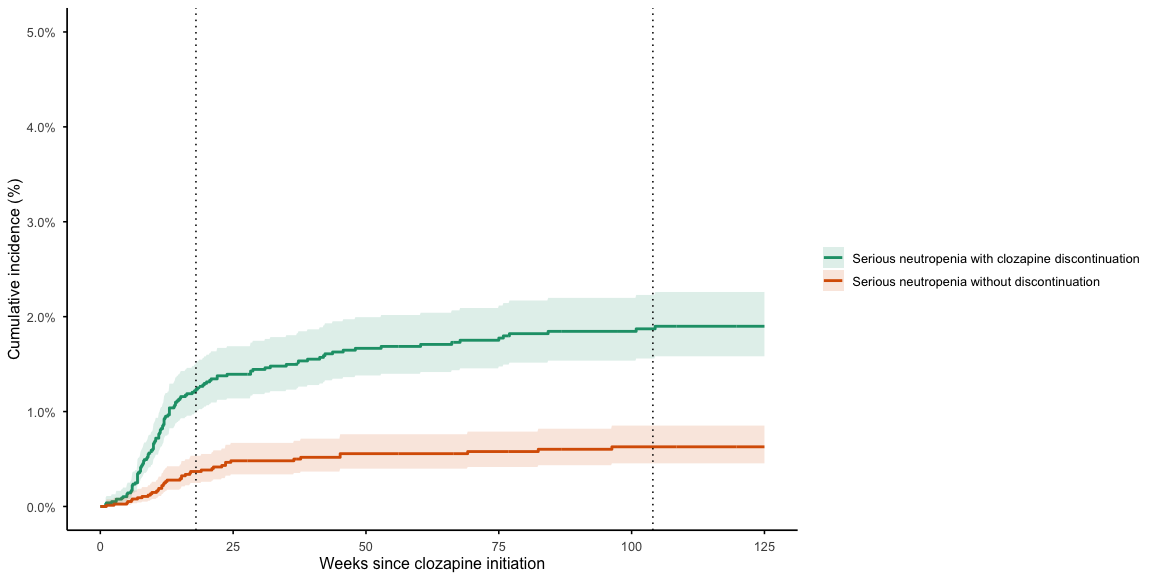
**

**Supplementary Figure 2: Cumulative incidence analysis for agranulocytosis.**

**
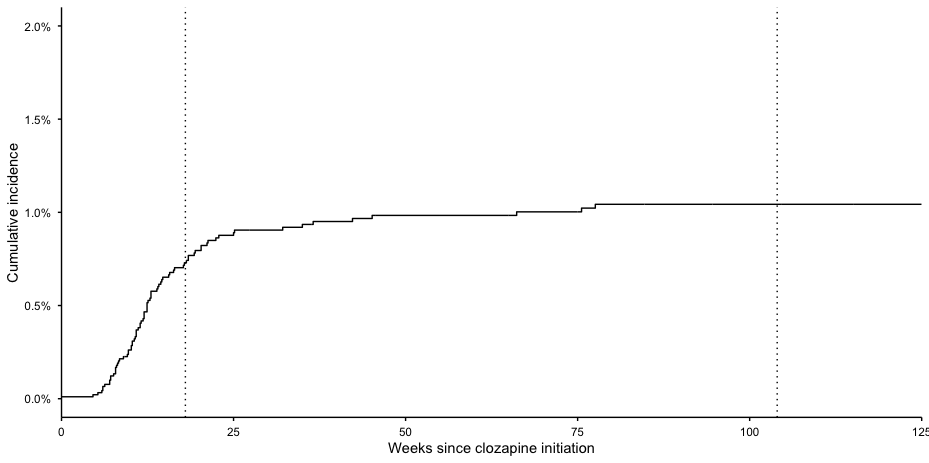
**

**Supplementary Table 1:** Annualised incidence rates for mild and serious neutropenia by ye**ar**

| Cohort | Event type | Incidence rate per 100 person years  (95% CI) | | |
| --- | --- | --- | --- | --- |
|  |  | Year1 | Year 2 | Year 3+ |
| Entire cohort | Any neutropenia | 5.24  (4.70-5.83) | 0.85  (0.61-1.16) | 0.45  (0.34-0.59) |
|  | Mild neutropenia | 4.41  (3.92-4.95) | 0.81  (0.57-1.11) | 0.40  (0.30-0.53) |
|  | Serious neutropenia | 2.35  (2.00-2.76) | 0.28  (0.15-0.47) | 0.15  (0.09-0.24) |
| Clozapine naïve | Any neutropenia | 5.31  (4.75-5.92) | 0.85  (0.60-1.16) | 0.46  (0.34-0.60) |
|  | Mild neutropenia | 4.44  (3.93-4.99) | 0.80  (0.56-1.11) | 0.41  (0.30-0.54) |
|  | Serious neutropenia | 2.45  (2.07-2.87) | 0.27  (0.14-0.47) | 0.16  (0.09-0.25) |
| Previous clozapine exposure | Any neutropenia | 3.95  (2.10-6.76) | 0.98  (0.12-3.53) | 0.28  (0.01-1.57) |
|  | Mild neutropenia | 3.95  (2.10-6.76) | 0.98  (0.12-3.53) | 0.28  (0.01-1.57) |
|  | Serious neutropenia | 0.61  (0.07-2.19) | 0.49  (0.01-2.72) | 0.00  (0.00-1.04) |

**Supplementary Table 2**: Full cumulative incidence analysis for mild neutropenia in people without previous clozapine exposure.

| Week post clozapine initiation | Cumulative incidence  (95% CI) | *n* events: *n* censored | *n* cumulative events:  *n* cumulative censored |
| --- | --- | --- | --- |
| 10 | 1.29% (1.06% - 1.57%) | 98:717 | 98:777 |
| 20 | 1.82% (1.53% - 2.15%) | 36:667 | 134:1444 |
| 30 | 2.16% (1.84% - 2.51%) | 21:490 | 155:1934 |
| 40 | 2.32% (1.98% - 2.69%) | 9:407 | 164:2341 |
| 50 | 2.41% (2.07% - 2.79%) | 5:308 | 169:2649 |
| 60 | 2.57% (2.21% - 2.97%) | 8:279 | 177:2928 |
| 70 | 2.65% (2.29% - 3.06%) | 4:284 | 181:3212 |
| 80 | 2.79% (2.41% - 3.21%) | 6:228 | 187:3440 |
| 90 | 2.81% (2.43% - 3.24%) | 1:232 | 188:3672 |
| 100 | 2.84% (2.45% - 3.26%) | 1:200 | 189:3872 |
| 110 | 2.94% (2.54% - 3.38%) | 4:171 | 193:4043 |
| 120 | 3.02% (2.62% - 3.47%) | 3:170 | 196:4213 |
| 130 | 3.08% (2.67% - 3.54%) | 2:155 | 198:4368 |
| 140 | 3.20% (2.77% - 3.68%) | 4:171 | 202:4539 |
| 150 | 3.20% (2.77% - 3.68%) | 0:162 | 202:4701 |
| 160 | 3.24% (2.80% - 3.72%) | 1:169 | 203:4870 |
| 170 | 3.27% (2.83% - 3.76%) | 1:115 | 204:4985 |
| 180 | 3.35% (2.90% - 3.84%) | 2:135 | 206:5120 |
| 190 | 3.43% (2.97% - 3.94%) | 2:106 | 208:5226 |
| 200 | 3.47% (3.00% - 3.99%) | 1:106 | 209:5332 |
| 210 | 3.51% (3.04% - 4.04%) | 1:131 | 210:5463 |
| 220 | 3.51% (3.04% - 4.04%) | 0:89 | 210:5552 |
| 230 | 3.56% (3.08% - 4.10%) | 1:107 | 211:5659 |
| 240 | 3.62% (3.13% - 4.16%) | 1:114 | 212:5773 |
| 250 | 3.67% (3.17% - 4.23%) | 1:103 | 213:5876 |
| 260 | 3.73% (3.22% - 4.30%) | 1:111 | 214:5987 |
| 270 | 3.86% (3.32% - 4.45%) | 2:86 | 216:6073 |
| 280 | 3.86% (3.32% - 4.45%) | 0:81 | 216:6154 |
| 290 | 3.93% (3.37% - 4.54%) | 1:114 | 217:6268 |
| 300 | 4.00% (3.43% - 4.64%) | 1:98 | 218:6366 |
| 310 | 4.00% (3.43% - 4.64%) | 0:94 | 218:6460 |
| 320 | 4.18% (3.57% - 4.87%) | 2:73 | 220:6533 |
| 330 | 4.18% (3.57% - 4.87%) | 0:78 | 220:6611 |
| 340 | 4.18% (3.57% - 4.87%) | 0:78 | 220:6689 |
| 350 | 4.18% (3.57% - 4.87%) | 0:81 | 220:6770 |
| 360 | 4.18% (3.57% - 4.87%) | 0:83 | 220:6853 |
| 370 | 4.48% (3.76% - 5.29%) | 2:73 | 222:6926 |
| 380 | 4.48% (3.76% - 5.29%) | 0:65 | 222:6991 |
| 390 | 4.48% (3.76% - 5.29%) | 0:78 | 222:7069 |
| 400 | 4.48% (3.76% - 5.29%) | 0:63 | 222:7132 |
| 410 | 4.48% (3.76% - 5.29%) | 0:47 | 222:7179 |
| 420 | 4.48% (3.76% - 5.29%) | 0:63 | 222:7242 |
| 430 | 4.48% (3.76% - 5.29%) | 0:23 | 222:7265 |
| 440 | 4.48% (3.76% - 5.29%) | 0:46 | 222:7311 |
| 450 | 4.48% (3.76% - 5.29%) | 0:23 | 222:7334 |
| 460 | 4.48% (3.76% - 5.29%) | 0:26 | 222:7360 |
| 470 | 4.48% (3.76% - 5.29%) | 0:24 | 222:7384 |
| 480 | 4.48% (3.76% - 5.29%) | 0:17 | 222:7401 |
| 490 | 4.48% (3.76% - 5.29%) | 0:23 | 222:7424 |
| 500 | 4.48% (3.76% - 5.29%) | 0:22 | 222:7446 |

**Supplementary Table 3**: Full cumulative incidence analysis for serious neutropenia in people without previous clozapine exposure.

| Week post clozapine initiation | Cumulative incidence  (95% CI) | *n* events: *n* censored | *n* cumulative events:  *n* cumulative censored |
| --- | --- | --- | --- |
| 10 | 0.80% (0.62% - 1.03%) | 60:717 | 60:777 |
| 20 | 1.68% (1.40% - 2.00%) | 60:667 | 120:1444 |
| 30 | 1.90% (1.60% - 2.24%) | 14:490 | 134:1934 |
| 40 | 2.04% (1.73% - 2.40%) | 8:407 | 142:2341 |
| 50 | 2.19% (1.86% - 2.56%) | 8:308 | 150:2649 |
| 60 | 2.21% (1.88% - 2.58%) | 1:279 | 151:2928 |
| 70 | 2.30% (1.96% - 2.68%) | 4:284 | 155:3212 |
| 80 | 2.36% (2.02% - 2.75%) | 3:228 | 158:3440 |
| 90 | 2.41% (2.06% - 2.81%) | 2:232 | 160:3672 |
| 100 | 2.44% (2.08% - 2.83%) | 1:200 | 161:3872 |
| 110 | 2.49% (2.13% - 2.89%) | 2:171 | 163:4043 |
| 120 | 2.49% (2.13% - 2.89%) | 0:170 | 163:4213 |
| 130 | 2.55% (2.18% - 2.96%) | 2:155 | 165:4368 |
| 140 | 2.58% (2.20% - 3.00%) | 1:171 | 166:4539 |
| 150 | 2.61% (2.23% - 3.03%) | 1:162 | 167:4701 |
| 160 | 2.68% (2.29% - 3.11%) | 2:169 | 169:4870 |
| 170 | 2.71% (2.32% - 3.15%) | 1:115 | 170:4985 |
| 180 | 2.71% (2.32% - 3.15%) | 0:135 | 170:5120 |
| 190 | 2.75% (2.35% - 3.20%) | 1:106 | 171:5226 |
| 200 | 2.84% (2.42% - 3.30%) | 2:106 | 173:5332 |
| 210 | 2.84% (2.42% - 3.30%) | 0:131 | 173:5463 |
| 220 | 2.84% (2.42% - 3.30%) | 0:89 | 173:5552 |
| 230 | 2.88% (2.46% - 3.36%) | 1:107 | 174:5659 |
| 240 | 2.94% (2.50% - 3.42%) | 1:114 | 175:5773 |
| 250 | 2.94% (2.50% - 3.42%) | 0:103 | 175:5876 |
| 260 | 2.99% (2.55% - 3.50%) | 1:111 | 176:5987 |
| 270 | 3.06% (2.60% - 3.58%) | 1:86 | 177:6073 |
| 280 | 3.06% (2.60% - 3.58%) | 0:81 | 177:6154 |
| 290 | 3.06% (2.60% - 3.58%) | 0:114 | 177:6268 |
| 300 | 3.06% (2.60% - 3.58%) | 0:98 | 177:6366 |
| 310 | 3.06% (2.60% - 3.58%) | 0:94 | 177:6460 |
| 320 | 3.06% (2.60% - 3.58%) | 0:73 | 177:6533 |
| 330 | 3.06% (2.60% - 3.58%) | 0:78 | 177:6611 |
| 340 | 3.06% (2.60% - 3.58%) | 0:78 | 177:6689 |
| 350 | 3.06% (2.60% - 3.58%) | 0:81 | 177:6770 |
| 360 | 3.06% (2.60% - 3.58%) | 0:83 | 177:6853 |
| 370 | 3.06% (2.60% - 3.58%) | 0:73 | 177:6926 |
| 380 | 3.22% (2.67% - 3.85%) | 1:65 | 178:6991 |
| 390 | 3.22% (2.67% - 3.85%) | 0:78 | 178:7069 |
| 400 | 3.22% (2.67% - 3.85%) | 0:63 | 178:7132 |
| 410 | 3.22% (2.67% - 3.85%) | 0:47 | 178:7179 |
| 420 | 3.22% (2.67% - 3.85%) | 0:63 | 178:7242 |
| 430 | 3.22% (2.67% - 3.85%) | 0:23 | 178:7265 |
| 440 | 3.22% (2.67% - 3.85%) | 0:46 | 178:7311 |
| 450 | 3.22% (2.67% - 3.85%) | 0:23 | 178:7334 |
| 460 | 3.22% (2.67% - 3.85%) | 0:26 | 178:7360 |
| 470 | 3.89% (2.64% - 5.50%) | 1:24 | 179:7384 |
| 480 | 3.89% (2.64% - 5.50%) | 0:17 | 179:7401 |
| 490 | 3.89% (2.64% - 5.50%) | 0:23 | 179:7424 |
| 500 | 3.89% (2.64% - 5.50%) | 0:22 | 179:7446 |

**Supplementary Table 4**: Full cumulative incidence analysis for mild neutropenia in people with previous clozapine exposure.

| Week post clozapine initiation | Cumulative incidence  (95% CI) | *n* events: *n* censored | *n* cumulative events:  *n* cumulative censored |
| --- | --- | --- | --- |
| 10 | 0.84% (0.46% - 1.44%) | 12:234 | 12:304 |
| 20 | 1.31% (0.81% - 2.03%) | 6:151 | 18:455 |
| 30 | 1.68% (1.08% - 2.50%) | 4:130 | 22:585 |
| 40 | 1.78% (1.16% - 2.62%) | 1:92 | 23:677 |
| 50 | 1.89% (1.24% - 2.77%) | 1:86 | 24:763 |
| 60 | 2.01% (1.33% - 2.92%) | 1:82 | 25:845 |
| 70 | 2.01% (1.33% - 2.92%) | 0:48 | 25:893 |
| 80 | 2.01% (1.33% - 2.92%) | 0:54 | 25:947 |
| 90 | 2.01% (1.33% - 2.92%) | 0:51 | 25:998 |
| 100 | 2.17% (1.44% - 3.16%) | 1:66 | 26:1064 |
| 110 | 2.17% (1.44% - 3.16%) | 0:39 | 26:1103 |
| 120 | 2.17% (1.44% - 3.16%) | 0:48 | 26:1151 |
| 130 | 2.17% (1.44% - 3.16%) | 0:28 | 26:1179 |
| 140 | 2.17% (1.44% - 3.16%) | 0:38 | 26:1217 |
| 150 | 2.44% (1.58% - 3.60%) | 1:28 | 27:1245 |
| 160 | 2.44% (1.58% - 3.60%) | 0:34 | 27:1279 |
| 170 | 2.44% (1.58% - 3.60%) | 0:27 | 27:1306 |
| 180 | 2.77% (1.76% - 4.14%) | 1:25 | 28:1331 |
| 190 | 2.77% (1.76% - 4.14%) | 0:24 | 28:1355 |
| 200 | 2.77% (1.76% - 4.14%) | 0:21 | 28:1376 |
| 210 | 2.77% (1.76% - 4.14%) | 0:21 | 28:1397 |
| 220 | 2.77% (1.76% - 4.14%) | 0:13 | 28:1410 |
| 230 | 2.77% (1.76% - 4.14%) | 0:13 | 28:1423 |
| 240 | 2.77% (1.76% - 4.14%) | 0:15 | 28:1438 |
| 250 | 2.77% (1.76% - 4.14%) | 0:17 | 28:1455 |
| 260 | 2.77% (1.76% - 4.14%) | 0:19 | 28:1474 |
| 270 | 2.77% (1.76% - 4.14%) | 0:9 | 28:1483 |
| 280 | 2.77% (1.76% - 4.14%) | 0:9 | 28:1492 |
| 290 | 2.77% (1.76% - 4.14%) | 0:15 | 28:1507 |
| 300 | 2.77% (1.76% - 4.14%) | 0:9 | 28:1516 |
| 310 | 2.77% (1.76% - 4.14%) | 0:7 | 28:1523 |
| 320 | 2.77% (1.76% - 4.14%) | 0:9 | 28:1532 |
| 330 | 2.77% (1.76% - 4.14%) | 0:10 | 28:1542 |
| 340 | 4.40% (1.83% - 8.73%) | 1:10 | 29:1552 |
| 350 | 6.50% (2.52% - 13.15%) | 1:7 | 30:1559 |
| 360 | 6.50% (2.52% - 13.15%) | 0:4 | 30:1563 |
| 370 | 6.50% (2.52% - 13.15%) | 0:7 | 30:1570 |
| 380 | 6.50% (2.52% - 13.15%) | 0:6 | 30:1576 |
| 390 | 6.50% (2.52% - 13.15%) | 0:8 | 30:1584 |
| 400 | 6.50% (2.52% - 13.15%) | 0:5 | 30:1589 |
| 410 | 6.50% (2.52% - 13.15%) | 0:5 | 30:1594 |
| 420 | 6.50% (2.52% - 13.15%) | 0:0 | 30:1594 |
| 430 | 6.50% (2.52% - 13.15%) | 0:0 | 30:1594 |
| 440 | 6.50% (2.52% - 13.15%) | 0:3 | 30:1597 |
| 450 | 6.50% (2.52% - 13.15%) | 0:0 | 30:1597 |
| 460 | 6.50% (2.52% - 13.15%) | 0:0 | 30:1597 |
| 470 | 6.50% (2.52% - 13.15%) | 0:0 | 30:1597 |
| 480 | 6.50% (2.52% - 13.15%) | 0:1 | 30:1598 |
| 490 | 6.50% (2.52% - 13.15%) | 0:0 | 30:1598 |
| 500 | 6.50% (2.52% - 13.15%) | 0:0 | 30:1598 |

**Supplementary Table 5**: Full cumulative incidence analysis for serious neutropenia in people with previous clozapine exposure.

| Week post clozapine initiation | Cumulative incidence  (95% CI) | *n* events: *n* censored | *n* cumulative events:  *n* cumulative censored |
| --- | --- | --- | --- |
| 10 | 0.21% (0.06% - 0.59%) | 3:234 | 3:304 |
| 20 | 0.37% (0.14% - 0.83%) | 2:151 | 5:455 |
| 30 | 0.37% (0.14% - 0.83%) | 0:130 | 5:585 |
| 40 | 0.47% (0.20% - 0.99%) | 1:92 | 6:677 |
| 50 | 0.47% (0.20% - 0.99%) | 0:86 | 6:763 |
| 60 | 0.47% (0.20% - 0.99%) | 0:82 | 6:845 |
| 70 | 0.60% (0.26% - 1.22%) | 1:48 | 7:893 |
| 80 | 0.60% (0.26% - 1.22%) | 0:54 | 7:947 |
| 90 | 0.60% (0.26% - 1.22%) | 0:51 | 7:998 |
| 100 | 0.60% (0.26% - 1.22%) | 0:66 | 7:1064 |
| 110 | 0.60% (0.26% - 1.22%) | 0:39 | 7:1103 |
| 120 | 0.60% (0.26% - 1.22%) | 0:48 | 7:1151 |
| 130 | 0.60% (0.26% - 1.22%) | 0:28 | 7:1179 |
| 140 | 0.60% (0.26% - 1.22%) | 0:38 | 7:1217 |
| 150 | 0.60% (0.26% - 1.22%) | 0:28 | 7:1245 |
| 160 | 0.60% (0.26% - 1.22%) | 0:34 | 7:1279 |
| 170 | 0.60% (0.26% - 1.22%) | 0:27 | 7:1306 |
| 180 | 0.60% (0.26% - 1.22%) | 0:25 | 7:1331 |
| 190 | 0.60% (0.26% - 1.22%) | 0:24 | 7:1355 |
| 200 | 1.01% (0.37% - 2.31%) | 1:21 | 8:1376 |
| 210 | 1.01% (0.37% - 2.31%) | 0:21 | 8:1397 |
| 220 | 1.01% (0.37% - 2.31%) | 0:13 | 8:1410 |
| 230 | 1.01% (0.37% - 2.31%) | 0:13 | 8:1423 |
| 240 | 1.01% (0.37% - 2.31%) | 0:15 | 8:1438 |
| 250 | 1.01% (0.37% - 2.31%) | 0:17 | 8:1455 |
| 260 | 1.01% (0.37% - 2.31%) | 0:19 | 8:1474 |
| 270 | 1.01% (0.37% - 2.31%) | 0:9 | 8:1483 |
| 280 | 1.01% (0.37% - 2.31%) | 0:9 | 8:1492 |
| 290 | 1.01% (0.37% - 2.31%) | 0:15 | 8:1507 |
| 300 | 1.01% (0.37% - 2.31%) | 0:9 | 8:1516 |
| 310 | 1.01% (0.37% - 2.31%) | 0:7 | 8:1523 |
| 320 | 1.01% (0.37% - 2.31%) | 0:9 | 8:1532 |
| 330 | 1.01% (0.37% - 2.31%) | 0:10 | 8:1542 |
| 340 | 1.01% (0.37% - 2.31%) | 0:10 | 8:1552 |
| 350 | 1.01% (0.37% - 2.31%) | 0:7 | 8:1559 |
| 360 | 1.01% (0.37% - 2.31%) | 0:4 | 8:1563 |
| 370 | 1.01% (0.37% - 2.31%) | 0:7 | 8:1570 |
| 380 | 1.01% (0.37% - 2.31%) | 0:6 | 8:1576 |
| 390 | 1.01% (0.37% - 2.31%) | 0:8 | 8:1584 |
| 400 | 1.01% (0.37% - 2.31%) | 0:5 | 8:1589 |
| 410 | 1.01% (0.37% - 2.31%) | 0:5 | 8:1594 |
| 420 | 1.01% (0.37% - 2.31%) | 0:0 | 8:1594 |
| 430 | 1.01% (0.37% - 2.31%) | 0:0 | 8:1594 |
| 440 | 1.01% (0.37% - 2.31%) | 0:3 | 8:1597 |
| 450 | 1.01% (0.37% - 2.31%) | 0:0 | 8:1597 |
| 460 | 1.01% (0.37% - 2.31%) | 0:0 | 8:1597 |
| 470 | 1.01% (0.37% - 2.31%) | 0:0 | 8:1597 |
| 480 | 1.01% (0.37% - 2.31%) | 0:1 | 8:1598 |
| 490 | 1.01% (0.37% - 2.31%) | 0:0 | 8:1598 |
| 500 | 1.01% (0.37% - 2.31%) | 0:0 | 8:1598 |

**Supplementary Table 6**: Competing risks regression analysis for mild and serious neutropenia with clozapine titration rate as an additional covariate (n=8070)

| **Covariate** | **Mild neutropenia** | | **Serious neutropenia** | |
| --- | --- | --- | --- | --- |
|  | **sHR**  **(95% CI)** | **p-value** | **sHR**  **(95% CI)** | **p-value** |
| Age (years) | 1.00  (0.99-1.01) | 0.460 | 1.05  (1.04-1.07) | <0.001 |
| Gender (male as the reference) | 0.76  (0.59-0.98) | 0.036 | 1.14  (0.85-1.53) | 0.380 |
| Baseline neutrophil count | 1.00  (1.00-1.00) | 1.000 | 1.00  (1.00-1.00) | 0.230 |
| Previous clozapine exposure (reference group: naïve) | 1.16  (0.65-2.09) | 0620 | 0.29  (0.09-0.91) | 0.035 |
| Clozapine titration rate (per 50mg increase at two weeks) | 1.05  (0.90-1.23) | 0.510 | 1.22  (1.02-1.45) | 0.027 |

**Supplementary Table 7**: Competing risks regression analysis for serious neutropenia with number of prior anti-psychotic medications as an additional covariate (n=7659)

| **Covariate** | **Serious neutropenia** | |
| --- | --- | --- |
|  | **sHR**  **(95% CI)** | **p-value** |
| Age (years) | 1.06  (1.04-1.07) | <0.001 |
| Gender (male as the reference) | 1.24  (0.91-1.69) | 0.170 |
| Baseline neutrophil count | 1.00  (1.00-1.00) | 1.000 |
| Previous clozapine exposure (reference group: naïve) | 0.45  (0.11-1.82) | 0.260 |
| Clozapine titration rate (per 50mg increase at two weeks) | 1.21  (1.01-1.45) | 0.042 |
| Number of antipsychotics at clozapine registration (per additional antipsychotic) | 1.21  (0.85-1.72) | 0.290 |
